# Patterns in the relationship between life expectancy and gross domestic product in Russia in 2005–15: a cross-sectional analysis

**DOI:** 10.1016/S2468-2667(19)30036-2

**Published:** 2019-04-04

**Authors:** Vladimir M Shkolnikov, Evgeny M Andreev, Rustam Tursun-zade, David A Leon

**Affiliations:** a Laboratory for Demographic Data, Max Planck Institute for Demographic Research, Rostock, Germany; b International Laboratory for Population and Health, Research University Higher School of Economics, Moscow, Russia; c Department of Non-communicable Disease Epidemiology, London School of Hygiene & Tropical Medicine, London, UK; d Department of Community Medicine, The Arctic University of Norway, Tromsø, Norway

## Abstract

**Background:**

Since 2005, Russia has made substantial progress, experiencing an almost doubling of per-capita gross domestic product by purchasing power parity (GDP [PPP]) to US$24 800 and witnessing a 6-year increase in life expectancy, reaching 71·4 years by 2015. Even greater gains in GDP (PPP) were seen for Moscow, the Russian capital, reaching $43 000 in 2015 and with a life expectancy of 75·5 years. We aimed to investigate whether mortality levels now seen in Russia are consistent with what would be expected given this new level of per-capita wealth.

**Methods:**

We used per-capita GDP (PPP) and life expectancy from 61 countries in 2014–15, plus those of Russia as a whole and its capital Moscow, to construct a Preston curve expressing the relationship between mortality and national wealth and to examine the positions of Russia and other populations relative to this curve. We adjusted life expectancy values for Moscow for underestimation of mortality at older ages. For comparison, we constructed another Preston curve based on the same set of countries for the year 2005. We used the stepwise replacement algorithm to decompose mortality differences between Russia or Moscow and comparator countries with similar incomes into age and cause-of-death components.

**Findings:**

Life expectancy in 2015 for both Russia and Moscow lay below the Preston-curve-based expectations by 6·5 years and 4·9 years, respectively. In 2015, Russia had a lower per-capita income than 36 of the comparator countries but lower life expectancy than 60 comparator countries. However, the gaps between the observed and the Preston-expected life expectancy values for Russia have diminished by about 25% since 2005, when the life expectancy gap was 8·9 years for Russia and 6·6 years for Moscow. When compared with countries with similar level of income, the largest part of the life expectancy deficit was produced by working-age mortality from external causes for Russia and cardiovascular disease at older ages for Moscow.

**Interpretation:**

Given the economic wealth of Russia, its life expectancy could be substantially higher. Sustaining the progress seen over the past decade depends on the ability of the Russian Government and society to devote adequate resources to people's health.

**Funding:**

This work was partly funded through the International Project on Cardiovascular Disease in Russia supported by a Wellcome Trust Strategic Award (100217) and was supported by the Russian Academic Excellence Project 5-100.

## Introduction

Mortality correlates with income across countries, with high-income countries usually having lower mortality than poorer countries. In 1975, this relationship was investigated by Samuel Preston in an article that was republished in 2007 with commentaries by the author and other scholars.^1^ In this influential study, the life expectancy at birth of countries around the world was found to be related to their gross national income per capita, a quantity that is frequently used as a marker of national wealth, living standards, and economic development. Preston showed that the cross-sectional relationship between life expectancy and per-capita national income across countries can be accurately described by the so-called Preston curve, with rapid increases in life expectancy in countries with lower incomes and slower increases in countries with higher incomes. Over time, the Preston curve tends to shift upwards (panel), reflecting improvements in life expectancy independent of national income (eg, due to adoption of medical advances).

Panel **The Preston curve shift**

The Preston curve is similar in shape to the logarithmic curve, with income along the *x*-axis and life expectancy along the *y*-axis. According to this formal relationship, the variation in life expectancy across countries per US$100 increase in per-capita national income is characterised by a steep increase in life expectancy among countries with low levels of income and a much slower increase in life expectancy among countries with high levels of national income.

A single Preston curve reflects a cross-sectional relationship in one period. Comparing these relationships between different time periods (1900s, 1930s, and 1960s) and taking into account differences in purchasing power, Preston found that the curves shifted upwards with time—ie, to higher life expectancies—with minor changes in shape. This pattern of change between periods suggested that the life expectancy increase over time was mostly attributable to the growing ability of the same amount of income to essentially buy more lifetime. Preston estimated that this accounted for 75–90% of the increase in life expectancy over time. This change was thought to operate through general improvements in knowledge about prevention and treatment diffusing across countries irrespective of levels of national income. The modern version of the Preston curve uses the per-capita GDP (PPP) as the measure of national wealth. More recent analyses have shown that the Preston relationship between life expectancy and per-capita GDP (PPP) has continued over the 1970s–2000s and still fits well with empirical data.^2^, ^3^, ^4^

The classic vertical shift of the Preston curve over time is visible even amid declines in national income. Notably, decreases in mortality rates in European countries were sustained between 2003 and 2012 despite the great economic recession of 2008 with the concomitant stagnation or decline in national wealth.^5^ In particular, Spain and Italy experienced persistently high longevity despite abrupt deteriorations in the labour market, which led to dramatic drops in the fraction of lifetime spent in employment.^6^, ^7^ More broadly, since the 1980s, life expectancy in many developing countries has increased substantially with little growth in national income. Furthermore, a recent Global Burden of Disease analysis^8^ has shown that mortality corresponding to the same level of their Socio-Demographic Index tends to decline with time.

Altogether, although these findings suggest that national income is an important resource for improvement of health, higher national wealth does not automatically result in improved health or increased survival.^9^ However, a study^4^ of European countries has found that over the past three decades, increases in life expectancy have been more closely associated with national wealth than in earlier periods. The authors suggest that this is because of the growing contribution of cutting-edge medical technologies to mortality decline whose adoption requires substantial increased investment in equipment and expertise.

GDP (PPP)=gross domestic product by purchasing power parity.

However, since the mid-1960s, Russian life expectancy and mortality patterns have differed strikingly from those in most industrialised countries that follow, on average, the Preston curve.^10^, ^11^ Russia has always stood out as being an exception to Preston's regular pattern. Paradoxically, between 1965 and 1984, the decrease in life expectancy in Russia and the Soviet Union coincided with growth in the income and wealth levels of the population.^12^ After a short-term improvement in life expectancy in 1985–87 that has been attributed to Gorbachev's anti-alcohol campaign, the health of the Russian population started to decline again and continued to do so throughout the 1990s, triggered by the abrupt return of easy access to alcohol and the economic and social stresses coinciding with the end of the Soviet Union.^10^, ^11^ These developments led to dramatic increases in mortality in 1992–94 and 1999–2003.^10^, ^11^ In the early 2000s, Russia had low levels of life expectancy and per-capita gross domestic product (GDP).^11^

**Research in context**

**Evidence before this study**

The Preston curve first published in 1975 describes the relationship between national life expectancy at birth and gross domestic product (GDP) per capita among countries. In 2001, we showed that life expectancy in Russia in 1995–96 was lower than was anticipated from the Preston curve. Over the period 2005–15, life expectancy and GDP per capita both increased substantially, leading to the question as to whether Russia's life expectancy was still less than might be expected given its per-capita GDP. We have regularly monitored the Russian and international literature in this field and were not aware of any other attempt to address this question.

**Added value of this study**

To our knowledge, this study is the first to show that, despite substantial improvements in national wealth per capita since 2005, Russia and its capital city, Moscow, remain outliers, underperforming in terms of life expectancy. Compared with other countries with similar levels of GDP per capita, the deficit in life expectancy is driven in part by excess mortality at working ages, whereas for Moscow, it is cardiovascular disease at older ages that is important.

**Implications of all the available evidence**

Russia needs to make better use of its wealth to improve the life expectancy of its population if its recent progress is to be sustained into the future. Investing more extensively and effectively in public health and primary prevention and addressing health inequalities will be crucial here.

In 2005–15, Russia's situation improved substantially: per-capita GDP by purchasing power parity (GDP [PPP]) doubled to US$24 800 and the country's life expectancy increased by 6 years to 71·4 years. The developments in the capital city of Moscow deserve particular attention. In both the communist and the post-communist eras, Muscovites benefited from higher wealth and lower mortality compared with the Russian population as a whole. In 2015, the city's per-capita GDP (PPP) was $43 000 (80% higher than Russia) and its life expectancy was 75·5 years (4·1 years higher than Russia).

In this Article, we investigate whether, after the improvements in 2005–15, Russia and its capital city remain outliers in the life expectancy–GDP (PPP) relationship defined by the Preston curve. We consider negative deviations below the curve as excess losses of human lifetime relative to those expected of countries with a given level of per-capita national wealth. Additionally, we seek to identify the major components of the life expectancy deficits in Russia and Moscow by decomposing the life expectancy differences by cause of death between these two populations on one side and countries with the same levels of per-capita GDP (PPP) on the other.

## Methods

### Populations and timepoints

We assessed the positions of Russia and Moscow at several timepoints according to average per-capita income and life expectancy relative to the broader international experience. We compare Moscow with Russia because, since the early 2000s, the capital city has had a much higher life expectancy and average income compared with the rest of Russia. We consider Russia as a whole rather than Russia without Moscow because our main focus is on Russia and we wanted to facilitate quantitative international comparisons and excluding Moscow, which comprises 8% of the national population, does not substantially change the Russian position (−0·4 years of life expectancy and −7% of per-capita GDP).

Although life expectancy began to increase in Russia after 2003,^10^, ^11^ in the first 2 years the positive changes were small and inconsistent across age groups. We have therefore taken 2005 as the earliest year for Russia and Moscow, when these trends had stabilised.

To build the Preston curve, we used data for 61 countries on life expectancy in 2005 and in 2015 (or 2014 where data were not available) and per-capita GDP (PPP) in 2005 and 2015 (measured in US dollars for the year in question). These countries comprise all those that have life tables computed by the direct method from vital statistics on age-specific deaths and population exposures.

### Data sources

The life expectancy data in 2014–15 for 61 countries other than Russia were extracted from the Human Mortality Database;^13^ the Eurostat Database;^14^ the Health for All Database;^15^ the database of the Pan American Health Organization;^16^ and the databases of the national statistical agencies of Canada,^17^ Singapore,^18^ and South Korea.^19^ The countries' per-capita GDP (PPP) values were derived from the World Bank.^20^ For Moscow, the comparable per-capita values were obtained using the regional GDP methodology by Zubarevich.^21^

Life expectancy for Russia and Moscow in 2005, 2010, and 2015 was calculated from the official mortality data by the Russian Statistical Service (Rosstat). Using our previous estimates,^22^ we calculated the life expectancy values for Moscow for these years, which were adjusted for underestimation of mortality at older ages. These estimates were lower than the original Rosstat life expectancy estimates by 0·2 years in 2005, 0·4 years in 2010, and 1·0 years in 2015.

Data on causes of death were extracted from the Russian Fertility and Mortality Database^23^ (Russia and Moscow in 2015) and from the WHO Mortality Database^24^ (Belgium, Croatia, Canada, Finland, Hungary, Poland, and the UK in 2014–15). Possible intercountry differences in coding practices are minimal at the level of the ten broad cause-of-death categories used in our decomposition analysis. Given an unexpected and supposedly artificial rise in deaths from senility in Russia after 2011,^25^ we take into account in our interpretation the possible overstatement of this ill-defined category at the expense of understatement of cardiovascular disease at ages 80 years and older in Russia and Moscow.

### Statistical analysis

To build the Preston curve, we use the simplest logarithmic form^3^

$${\text{LE}}_{i}=a+b\cdot \log({\text{GDP}}_{i})+{ɛ}_{i}$$

where index *i* indicates the country, ɛ_i_ is the random error specific to that country, and *a* and *b* are parameters to be estimated. Calculations of the parameters and standard statistics to assess model fit (*R*^2^ and *F*-test) were done using the command *curve estimation* in SPSS. For our modelled curves, we found that 68–71% of the life expectancy variance could be explained by the per-capita GDP, with high statistical significance of the relationship (p<0·0001). We observed no pattern in the regression residuals plotted against the fitted values.

The deviations of Russia and Moscow from the Preston curve were calculated as differences between the GDP-based life expectancy values obtained from our logarithmic model and the observed values of life expectancy in those years.

Although earlier studies have examined mortality and life expectancy differences between Russia and national populations with much higher levels of income such as France and the UK,^10^, ^11^ the present study decomposes, by age and causes of death, the differences in life expectancy between countries with the same levels of per-capita GDP as Russia or Moscow. For Russia, these countries are Romania, Croatia, Hungary, and Poland, whereas for Moscow, they are the UK, Finland, Canada, and Belgium. We identified these countries by choosing four countries that had the closest per-capita GDP (PPP) to Russia or Moscow, a similar level of Socio-Demographic Index, and were positioned closer to the Preston curve.

Decomposition of a difference between two life expectancies is a demographic method to assess the contributions of different age groups and causes of death (within these groups) to the total life expectancy differences between two populations. In this study, decompositions of life expectancy gaps between Russia and Moscow on one side and the eight comparator countries on the other side were done with the stepwise replacement algorithm.^26^ This general method executes a chain of replacements of elementary age-cause-specific death rates in one population by corresponding rates from another population and vice versa and calculates life expectancy effects after each replacement.

We used SPSS version 25.0 to fit the regression model and Excel for life expectancy decompositions.

### Role of the funding source

The funders of the study had no role in study design; data collection, analysis, or interpretation; or writing of the report. The authors had full access to all the data in the study and the corresponding author had final responsibility for the decision to submit for publication.

## Results

**Figure 1:**
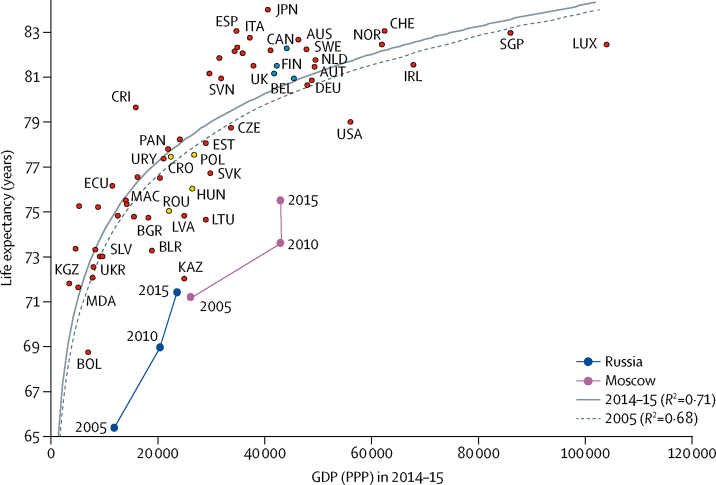
Life expectancy and GDP (PPP) for 61 nations for the 2014–15 period, the Preston curves for 2005 and 2014–15, and the positions of Russia and Moscow in 2005, 2010, and 2015 The figure shows the Preston curve for 2005 (dashed) and for 2014–15 (solid). Comparator countries for Russia are marked in yellow and comparator countries for Moscow are marked in blue. AUS=Australia. AUT=Austria. BEL=Belgium. BGR=Bulgaria. BLR=Belarus. BOL=Bolivia. CAN=Canada. CHE=Switzerland. CRI=Costa Rica. CRO=Croatia. CZE=Czech Republic. DEU=Germany. ECU=Ecuador. ESP=Spain. EST=Estonia. FIN=Finland. HUN=Hungary. IRL=Ireland. ITA=Italy. JPN=Japan. KAZ=Kazakhstan. KGZ=Kyrgyzstan. LUX=Luxemburg. LTU=Lithuania. LVA=Latvia. MAC=Macedonia. MDA=Moldova. NLD=Netherlands. NOR=Norway. PAN=Panama. POL=Poland. ROU=Romania. SGP=Singapore. SLV=El Salvador. SVK=Slovakia. SVN=Slovenia. SWE=Sweden. UKR=Ukraine. URY=Uruguay. GDP (PPP)=gross domestic product by purchasing power parity in US dollars.

Between 2005 and 2015, Russia experienced rapid growth in per-capita GDP (PPP) combined with a gain of 6 additional years of life expectancy. Most of this GDP growth occurred between 2005 and 2010, with almost no improvement in the following 5 years (figure 1). The rise in life expectancy continued in 2011–15, albeit at a slower pace. A similar trajectory, but at higher levels of GDP (PPP) and life expectancy, was observed for Moscow. In terms of both GDP (PPP) and life expectancy, the levels for Moscow in 2005 were close to the levels for Russia in 2015. In 2005–10, per-capita GDP (PPP) in Moscow grew by 65% and reached levels similar to those of several established market economies. Between 2010 and 2015, per-capita GDP (PPP) in Moscow did not rise.

Despite this progress, life expectancy for both Russia and Moscow in 2005 and 2015 was well below the line of the Preston curve (figure 1). In 2005, the difference between the observed and the expected life expectancy values was 8·9 years for Russia and 6·6 years for Moscow; in 2015, these differences decreased to 6·5 years for Russia and 4·9 years for Moscow. For Russia, these differences were the largest negative deviations among all countries considered in both years, whereas for Moscow they were the fourth and third largest negative deviations, respectively. In 2014–15, Russia's per-capita GDP (PPP) was nearly the same as in Croatia, Romania, Uruguay, Hungary, and Poland, all of which had appreciably higher life expectancy (figure 1). Over the same period, Russia's life expectancy was similar to that of Kyrgyzstan and Moldova and was slightly lower than that of Ukraine, El Salvador, and Belarus, where the per-capita GDP (PPP) values were lower than those in Russia.

In 2015, Russia was positioned close to Kazakhstan in the life expectancy–GDP space, but Moscow had no close neighbour (figure 1). Although Moscow had a per-capita GDP (PPP) level similar to that of Finland, the UK, Belgium, Canada, and Italy, it lagged behind these countries in life expectancy by 5–7 years. The life expectancy of the Moscow population was similar to that of Latvia, Macedonia, Hungary, and Ecuador, all of which had a per-capita GDP (PPP) lower than Moscow.

Between 2005 and 2015, the differences between the observed and the expected life expectancy values diminished by approximately 25% (by 2·3 years for Russia and 1·7 years for Moscow). The vertical or almost vertical moves in the 2011–15 period for both Moscow and Russia reduced but did not eliminate the negative vertical deviations from the Preston curve. In these years, life expectancy was increasing with stagnating or even diminishing national income. Thus, both Moscow and Russia as a whole moved to be somewhat more in line with the other countries.

**Figure 2:**
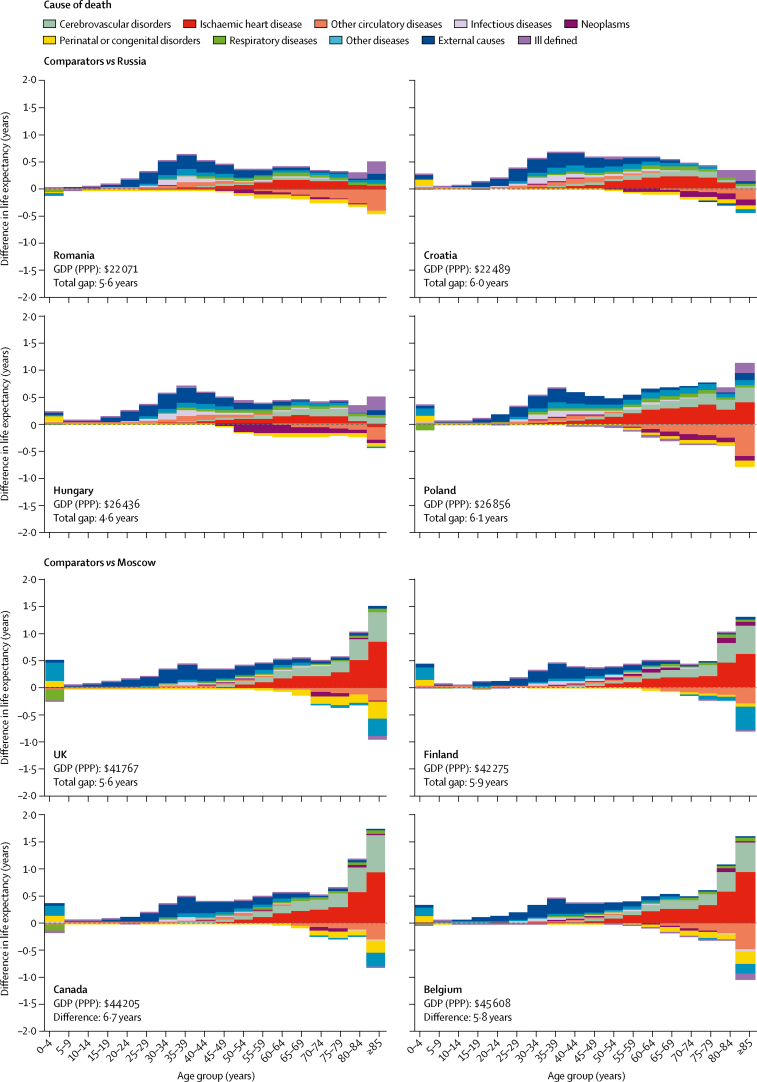
Age-cause-specific components of the life expectancy gaps between Russia or Moscow and international comparators in 2014–15 Each panel shows age-cause-specific contributions to the total differences in life expectancy between Russia or Moscow and the countries with the closest average per-capita income. The four upper panels show results of decompositions of the gaps between four countries and Russia in 2014–15 whereas the four bottom panels show similar decompositions for Moscow. Each panel shows vertical bars corresponding to contributions of mortality differences within 5-year age groups to the total life expectancy difference. Coloured parts within each vertical bar correspond to contributions of differences in mortality from selected causes within the age groups to the total life expectancy difference. GDP (PPP)=gross domestic product by purchasing power parity in US dollars.

In 2015, the four countries with average incomes closest to Russia were in eastern Europe; all four lay below the Preston curve, with distances from the curve varying from 0·2 years in Hungary to 2·5 years in Romania. The life expectancy gaps between Russia and these comparator countries varied from 4·6 years (Hungary *vs* Russia) to 6·1 years (Poland *vs* Russia; figure 2).

For each of the four comparator countries, the breakdown of the life expectancy gap with Russia was similar in terms of the distribution of the age and cause components (figure 2). The greatest contributions were produced by differences in mortality at working ages (ie, 15–64 years), mostly from the increased mortality from external causes in Russia. Smaller but substantial contributions were produced by mortality differences at older ages, mostly from ischaemic heart disease and cerebrovascular disorders. The summary contribution of the Russian mortality excess at all ages younger than 60 years was 2·2 times (Poland) to 4·5 times (Romania) greater than the contribution of all ages older than 60 years.

In 2015, the comparator countries for Moscow were established market economies of western Europe and North America. The four comparator countries lay above the Preston curve with the vertical distances varying from 0·1 years (Belgium) to 1·5 years (Canada; figure 1). The gaps from Moscow vary from 5·4 years (Belgium *vs* Moscow) to 6·7 years (Canada *vs* Moscow; figure 2). Here, a major role is played by the excess in cardiovascular mortality at older ages in Moscow, with smaller but substantial contributions of external causes at working ages. The components produced by the excess mortality of Muscovites at all ages older than 60 years were nearly the same as components produced by all ages younger than 60 years.

In all eight comparisons, the excess in mortality at older ages (especially at ages 85 and older) in Russia and Moscow from ischaemic heart disease and cerebrovascular disorders was partly counterbalanced by opposite contributions related to the group of other circulatory diseases and other causes of death (figure 2); these findings might result from peculiarities of Russia in diagnostics and coding of causes of death among the older population, as acknowledged in the Methods.

## Discussion

This study examined the relationship between life expectancy and per-capita GDP in Russia and its much wealthier capital city Moscow in comparison with 61 other countries by means of the Preston curve. Russia and Moscow's large negative deviations from the curve in 2015 can be regarded as estimates of losses of human life that could, in principle, have been avoided if Russia's considerable national wealth were more effectively orientated towards improving the population's health.

This study highlights the rapid economic growth in Russia that had, by 2015, allowed the country to overtake many eastern European nations in terms of per-capita GDP. Moscow has benefited especially, with a recent per-capita GDP comparable to that of many established market economies. Although life expectancy did not always increase with national income, economic growth in Russia in 2005–10 was accompanied by a rise in health expenditure and general living standards and thus by a substantial increase in life expectancy.^11^ Our results have also shown that since 2010, life expectancy continued to increase even though per-capita GDP stagnated. This movement could be viewed as following the classic upwards shift that was first described by Preston's curves from different epochs. It suggests that after four decades of predominant mortality increase, including the acute crisis of the 1990s, Russia is finally improving survival and that the lifestyles and circumstances of the Russian population are changing for the better.

However, in spite of this progress, life expectancy in Russia and in Moscow in 2015 still remains well below the estimate offered by the Preston curve, by 6·5 years for Russia and 4·9 years for Moscow. Russia has lower per-capita GDP (PPP) than 36 of our considered countries, whereas it has lower life expectancy than 60 of those countries—all except for Bolivia. Meanwhile, Moscow has lower per-capita GDP (PPP) than only 14 countries but has lower life expectancy than 41 countries. Closing the 6·5-year gap for Russia will take time: according to the Human Mortality Database,^13^ when the UK's life expectancy was at the same level as Russia's current life expectancy, it took the UK 34 years (from 1966 to 2000) to gain 6·5 years of lifetime. Strikingly, Estonia made such a gain in just 15 years (2001–16), illustrating that with modern approaches to health care and prevention, such improvements are possible in post-Soviet countries with high starting levels of mortality over relatively short periods.

Our study throws light on the specific causes of death that underlie the deficit in life expectancy seen in Russia relative to countries with similar levels of GDP per capita. Figure 2 suggests that each of the comparator countries differs markedly from either Russia or Moscow in terms of mortality structure by age and cause. However, the ways in which they deviate from Russia or Moscow are very similar, underlining that Russia and Moscow are distinctive outliers for given levels of national wealth not just in terms of level of mortality, but also in terms of age and cause structure of mortality.

The deficit in life expectancy relative to countries with similar levels of GDP per capita is mainly explained by high mortality from external causes at working ages and from cardiovascular disease at older ages. The first is particularly important in the comparisons between Russia and central and eastern European countries, whereas the second is more pronounced in comparisons between Moscow and western countries.

There are many factors that underlie high external cause and cardiovascular mortality in Russia. Behavioural factors include a high prevalence of hazardous alcohol consumption and male smoking. Smoking increases risk of death from a wide range of conditions from cancers to cardiovascular disease. Similarly, alcohol drinking has widespread effects on survival as a consequence of injuries and violence, but also through its effect on risk of premature deaths from heart attacks and stroke.^27^, ^28^ In Moscow and other regions, alcohol-related and external cause mortality has decreased substantially. Alcohol policy measures implemented in 2005–07, which included stricter control of alcohol manufacture, processing, and sale, were followed by substantial drops in mortality.^10^ This result is consistent with evidence that after the mid-2000s, the Russian population experienced decreases in consumption of vodka, although the present levels are still high.^29^ The other positive development is a reduction in male smoking that has been apparent since around 2010,^30^, ^31^ although once again it is still much higher than in other high-income countries.

An adverse profile of drinking and smoking can only explain part of the life expectancy deficit, particularly among women who have historically had lower prevalence of smoking and heavy drinking. We have previously shown that there is substantial inter-regional variation in cardiovascular mortality across Russia, which is the main reason for little change in inter-regional variation in life expectancy in the 2000s and 2010s.^32^ Part of this regional difference in cardiovascular mortality is likely to reflect variations in the ability of regional health systems to reduce the burden of cardiovascular disease, which requires modernisation of detection and treatment in primary care as well as investment in modern diagnostic and interventional technologies. These differences might be exacerbated by regional differences in levels of financing of the medical care system including exceptionally favourable positions of Moscow and a few other regions. Nevertheless, since 2010, Russia has experienced an important rise in the availability of cardiovascular surgery and centres capable of carrying out emergency percutaneous coronary interventions.^10^, ^33^ However, in Russia overall, per-capita expenditure on health—including public health—remains lower than in most neighbouring countries.^34^ One of the more alarming features of the low level of investment in and effectiveness of public health in Russia is the alarming ten-fold growth in deaths from HIV/AIDS from 2005 to 2015. For the time being, this serious situation has minimal impact on life expectancy as deaths from HIV/AIDS account for only 1% of all deaths in 2015.

Other important factors behind the life expectancy deficit in Russia are related to human insecurity (ie, technical and organisational measures to prevent fatal accidents), weaknesses in policing and enforcement, and suboptimal safety standards. In synergy with alcohol, these factors elevate risks of fatal injuries and violent death. The current level of mortality from these causes suggests that the financial resources allocated to addressing these challenges are still insufficient or are being used ineffectively. It is well established that high levels of income inequality are related to lower life expectancy.^35^, ^36^, ^37^ In line with Russia's pronounced socioeconomic inequality and related social stress, there are large gaps in the mortality levels and trends of the better-off and the worse-off population groups in Russia. It has been previously shown that this inequality is indeed slowing Russia's progress towards better health.^38^, ^39^

At the most general level, the issue of inadequate government investment in policies and actions to improve human health, safety, and welfare might tie in with the fact that in Russia, a high share of GDP comes from extractive industries. It has been shown that countries that depend heavily on the mining sector tend to spend a smaller proportion of their wealth on health and education than do other countries and to have life expectancies that lie substantially below the Preston curve.^40^

Finally, in Russia there is a common belief that the country's poor health and high mortality are to be considered natural and inevitable because of the economic backwardness of the country. Thus, Russians often blame their poor health and high mortality on Russia's poverty. Our study shows that this perception is wrong because Russian mortality is far higher than it should be given the level of national wealth per capita. Put simply, the economic wealth of Russia is more than sufficient to allow its population to have a higher level of life expectancy.

Our study had limitations. We were restricted to a set of 61 countries chosen because of their reliable mortality statistics. Therefore, the data cover only a part of the world's population, with a strong bias towards European, North American, central Asian, and Latin American regions that are much closer to Russia with respect to their epidemiological profiles than the Indian subcontinent or Africa. However, inclusion of these regions if the data had been of adequate quality would be unlikely to affect Russia's position relative to the Preston curve compared with the comparator countries that we did analyse. In addition, mortality at ages 80 years or older is understated in both Russia and Moscow, meaning that our data risked underestimating the life expectancy gap. However, for the whole of Russia, this data shortcoming does not produce visible changes in life expectancy at birth; for Moscow, we adjusted life expectancy values for old-age mortality understatement. As usual, international cause-of-death comparisons are problematic because of possible inconsistency of diagnostics and coding practices across countries. However, we minimised this problem by using only ten very broad diagnostic categories. It is, nonetheless, still possible that at ages 80 years and older, Russian mortality from ill-defined and some other causes is overstated whereas mortality from cardiovascular diseases is somewhat understated.

In conclusion, thanks to the recent progress outlined in this Article, Russia has reduced the distance of its life expectancy from the value predicted by the Preston curve by about a quarter but remains considerably below it. Despite this positive trend, future developments are hard to predict given the long legacy of neglect and under-financing of the Russian health system. In this context, it is particularly worrying that since 2012, increases in the Russian health expenditure have been slowing down and the federal state budget's part of this expenditure (ie, the most reliable part) has been shrinking.^41^, ^42^ The sustainability of current levels of progress towards the Preston curve depends on the willingness of Russian leadership and society to devote adequate attention as well as resources to the country's health.
